# Phylogeography of two closely related species of *Allium* endemic to East Asia: Population evolution in response to climate oscillations

**DOI:** 10.1002/ece3.4338

**Published:** 2018-07-16

**Authors:** Jingtian Yang, Songdong Zhou, Deqing Huang, Xingjin He

**Affiliations:** ^1^ Key Laboratory of Bio‐Resources and Eco‐Environment of Ministry of Education College of Life Sciences Sichuan University Chengdu China

**Keywords:** *Allium*, central and northern China, climate oscillations, cpDNA, phylogeography, species divergence

## Abstract

This study investigated the effects of climate oscillations on the evolution of two closely related *Allium* species, *A. neriniflorum* and *A. tubiflorum*. We sequenced three cp DNA (cpDNA) fragments (*rps*16, *rpl*32‐*trn*L, and *trn*D‐*trn*T, together approximately 2,500 bp in length) of two closely related *Allium* species, with samples from 367 individuals in 47 populations distributed across the total range of these species. The interspecific and intraspecific divergence times of the two species were in the Quaternary glaciation. The population divergence was high for the cpDNA variation, suggesting a significant phylogeographic structure (*N*_ST_ = 0.844, *G*_ST_ = 0.798, *p* < 0.05). Remarkable ecological differentiation was also revealed by Niche models and statistical analyses. Our results suggest the speciation event of the two species was triggered by violent climatic changes during the Quaternary glaciation.

## 1 INTRODUCTION

Climatic oscillations and related changes in vegetation can be recognized in the period from 3.6 to 0.8 Ma (Naidina & Richards, 2016). Multiple studies have showed that the climate oscillations especially in the glaciation cycles of the Quaternary may have facilitated speciation, diversification, hybridization, and changes in the distribution of the global vegetation (Liu et al., 2018; Shahzad, Jia, Chen, Zeb, & Li, 2017; Yamamoto, Ohtani, Kurata, & Setoguchi, 2017).

Although East Asia was profoundly affected by global climatic oscillations, even in the Quaternary ice ages, no massive ice sheet development occurred in this region (Hewitt, 2000). Therefore, East Asia was the most important refuge for many species during these climate fluctuations (Qiu, Fu, & Comes, 2011). Fossil‐based biome reconstructions of East Asian species predict that, during the last glacial maximum (LGM), the temperate forests that currently dominate East Asia retreated southward to 25°N to 30°N (Harrison, Yu, Takahara, & Prentice, 2001). Nevertheless, recent phylogeographic studies have highlighted in situ survival of hardy alpine herbs and forest trees during glacial periods in northern China. Many refugia scenarios have been revealed for these species, such as single refugium (Liu & Harada, 2014), multiple refugia (Tian et al., 2009), refugia within refugia (Wang, Gao, Kang, Lowe, & Huang, 2009), cryptic refugia, or microrefugia (Stewart, Lister, Barnes, & Dalen, 2010).

Orographic influences have also had a crucial role in the inter–intraspecific divergence and population demography of speciation. Recently, many phylogeographic studies have assumed a causal link between geological processes (orogenesis) and biological responses (diversification) (Chen et al., 2012; Jiang, Zhang, Zhang, & Sanderson, 2014; Wang & Yan, 2014; Wang, Zhang, & Yin, 2016). The results of these studies indicate that most of the mountain taxa show high levels of biodiversity. In contrast to a single causal link between geological processes and biological responses, Mosbrugger, Favre, Muellner‐Riehl, Päckert, and Mulch (2018) provided the “mountain‐geo‐biodiversity hypothesis”. This hypothesis suggests that not only surface uplift, but full elevational zonation, which provides both refugia for the persistence of lineages during climate modifications and geographic barriers, are vital to promote allopatric speciation. The study of Hauenschild et al. (2017) about the widespread genus Allium also supported this hypothesis.

*Allium* L., one of the largest monocotyledon genera, is widely distributed in northern temperate zones such as the Eastern Mediterranean Sea, Southwest Asia, Central Asia, and East Asia. There are more than 900 species in the world, of which 140 species (51 endemic species and five imported species) are in China. Most species of *Allium* have an onion flavor and adapt to arid climatic conditions with developed underground storage organs. However, it is very difficult to resolve *Allium* taxonomy due to the great diversity in the morphology, biological learning, and ecological adaptation of *Allium* (Li et al., 2010). Sect. Caloscordum, a subgenus in *Allium,* is unique, as it lacks the smell of garlic and perianth segments united into a tube proximally. Three species in Sect. Caloscordum were recorded in the flora of China, namely, *Allium neriniflorum* (Herbert) G. Don (*A. neriniflorum*), *Allium tubiflorum* Rendle (*A. tubiflorum*), and *Allium inutile* Makino (*A. inutile*) (Xu & Kamelin, 2000). *Allium neriniflorum* is located in the northern and northeastern parts of China, far east Russia and Mongolia. *Allium tubiflorum* is mainly distributed in the central and northern areas of China. *Allium inutile* is just located in Chu Xian (Anhui, China) and Japan (Xu & Kamelin, 2000).

According to flora of china, we went to collect samples of three species in Sect. Caloscordum in the field. However, during our field sampling, we just found *A. neriniflorum* and *A. tubiflorum* and did not find *A. inutile*. Previous studies investigating *A. neriniflorum* and *A. tubiflorum* have shown that the two species are sister groups and have a close relationship (Li & Xu, 1996; Lu, Yang, Lu, Zhou, & He, 2016). The leaf micromorphology characters of *A. neriniflorum* and *A. tubiflorum* are different, which can serve as a classification basis (Lu et al., 2016).

In this study, we sampled populations across the distribution range of *A. neriniflorum* and *A. tubiflorum* for phylogeographic analyses and used three sets of cpDNA fragment sequences to construct phylogeographic patterns for these two species. We aimed to answer the following three questions: (a) Are *A. neriniflorum* and *A. tubiflorum* significantly different from molecular levels? Is the genetic diversity of *A. neriniflorum* and *A. tubiflorum* analogous? (b) Where were the refugia of both species during the climatic oscillations? Did they survive in situ or retreat to somewhere below to 30°N during climatic oscillations? (c) How did the climatic oscillations or other geological factors effect on the population evolutionary history of these two species?

## 2 MATERIALS AND METHODS

### 2.1 Population sampling

**Figure 1:**
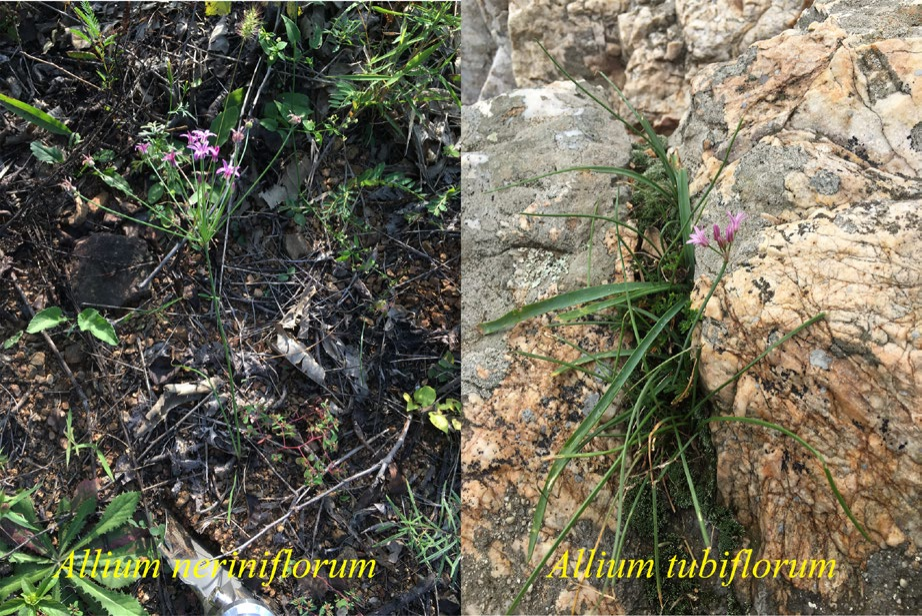
Photograph showing the phenotypic differences between *Allium neriniflorum* and *Allium tubiflorum*

According to flora of china, we went to collect samples of three species in Sect. Caloscordum in the field. However, during our field sampling, we did not find *A. inutile* in Anhui, China. As for *A. neriniflorum* and *A. tubiflorum*, we found that their morphological characters are very similar. According to our statistical analysis, the main difference in the shape of these two species based on quantitative characters is that the plant height and pedicel length, *A. neriniflorum* are usually taller in plant height and longer in pedicel length than *A. tubiflorum* (see Figure 1). We also found that the ecological conditions of *A. neriniflorum* and *A. tubiflorum* differ. *A. neriniflorum* is easily found in damp places and meadows of low altitude. In contrast, *A. tubiflorum* mainly grows in the rock crevices of high‐altitude mountains. The ecological boundary conditions of these species is in the northern edge of Taihang Mountain at 36.97°N.

**Figure 2:**
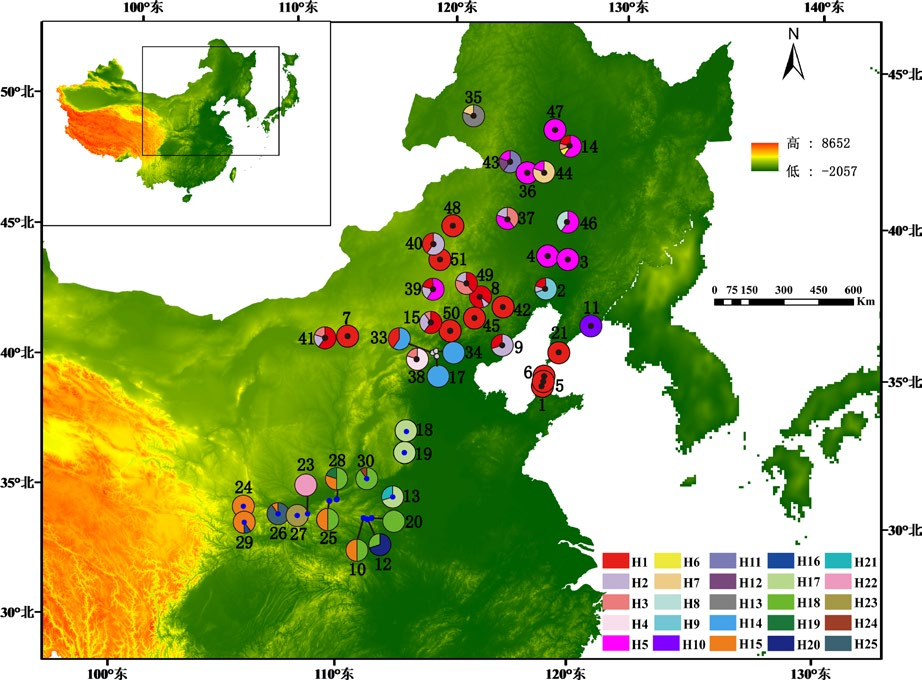
Map of the sampling locations and the geographic locality of the cpDNA haplotypes detected for *Allium tubiflorum* and *Allium neriniflorum*. Black circles indicate the distribution of *A. neriniflorum* populations; blue circles represent the *A. tubiflorum* populations. In addition, the white circles represent probable hybrid populations. The cpDNA haplotypes (H1–H25) and their frequencies in each population are indicated by the colorful pie charts. The population codes and information are shown in Table 1

Healthy leaves from *A. tubiflorum* and *A. neriniflorum* were collected and dried by silica gel for DNA extraction until DNA extraction. Each individual no closer than 50 m from the nearest next nearest individual. Forty‐seven populations with 4–13 individuals each were sampled in total, covering all the distribution regions of *A. neriniflorum* (30 populations, 204 individuals), *A. tubiflorum* (14 populations, 140 individuals), and three probable hybrid populations (23 individuals) in China (Table 1, Figure 2). Voucher specimens (Table 1) were also collected for all sampled populations. The specimens were deposited in the archives of the Herbarium of Sichuan University (SZ).

**Table 1:** Information of sample location and sample size of *Allium tubiflorum*,* Allium neriniflorum* and Hybrid

Pop. code	Species	Location	Latitude (N)	Longitude (E)	Elevation (m)	*N*	Haplotypes (no. of individuals)	Endemic haplotypes	*H* (gene diversity)	*π* (nucleotide diversity)
1	*A. neriniflorum*	Changdao, ShanDong	37°59′	120°41′	223	10	H1(10)	0	0	0
2	*A. neriniflorum*	Yiwulvshan, LiaoNing	41°36′	121°42′	318	10	H1(2), H9(7), H2(1)	1	0.511	0.00116
3	*A. neriniflorum*	Bahushan, LiaoNing	42°30′	123°12′	233	10	H5 (10)	0	0	0
4	*A. neriniflorum*	Daqinggou, IMG	42°47′	122°10′	246	10	H5 (10)	0	0	0
5	*A. neriniflorum*	Tuoji, ShanDong	38°10′	120°44′	124	4	H1(4)	0	0	0
6	*A. neriniflorum*	Nanhuang, ShanDong	38°22′	120°53′	278	5	H1(5)	0	0	0
7	*A. neriniflorum*	Baishitougou, IMG	40°47′	111°27′	1,181	10	H1(10)	0	0	0
8	*A. neriniflorum*	Wangyedian, IMG	41°43′	118°22′	956	10	H1(9), H2(1)	0	0.2	0.00052
9	*A. neriniflorum*	Jieshishan, HeBei	39°45′	119°08′	533	10	H1(3), H2(7)	0	0.467	0.0016
11	*A. neriniflorum*	Dagushan, LiaoNing	39°54′	123°35′	153	10	H10 (10)	1	0	0
14	*A. neriniflorum*	Lamadianzi, HeiLongjiang	46°42′	124°39′	135	10	H1(2), H5 (6), H3(1), H6(1)	1	0.644	0.002
15	*A. neriniflorum*	Jingeshan, HeBei	41°00′	115°44′	1,176	10	H1(6), H2(3), H3 (1)	0	0.6	0.00189
21	*A. neriniflorum*	Daheishan, LiaoNing	39°06′	121°46′	280	10	H1(10)	0	0	0
35	*A. neriniflorum*	Yimin, HeiLongjiang	48°34′	119°46′	673	5	H13(4), H7(1)	1	0.4	0.00121
36	*A. neriniflorum*	Wulanhaote, IMG	46°01′	122°04′	304	5	H8 (5)	0	0	0
37	*A. neriniflorum*	Hanshan, IMG	44°27′	120°30′	584	5	H2(1), H3 (2), H5 (2)	0	0.8	0.00199
38	*A. neriniflorum*	Caogoubao, HeBei	39°40′	114°49′	1,490	5	H3 (1), H4(4)	1	0.4	0.00138
39	*A. neriniflorum*	Zhenglanqi, IMG	42°16′	116°03′	1,292	5	H1(1), H2(1), H5 (3)	0	0.7	0.00155
40	*A. neriniflorum*	Baiyinxile, IMG	44°00′	116°24′	1,177	5	H1(2), H2(3)	0	0.6	0.00206
41	*A. neriniflorum*	WuDangzhao, IMG	40°47′	110°18′	1,522	5	H1(3), H2(1), H3 (1)	0	0.7	0.00211
42	*A. neriniflorum*	Jinhuashan, LiaoNing	41°11′	119°28′	561	5	H1(5)	0	0	0
43	*A. neriniflorum*	Suolun, IMG	46°37′	121°15′	490	5	H5(1), H12 (1), H11 (3)	2	0.7	0.0006
44	*A. neriniflorum*	Beidagang, JiLin	46°00′	122°59′	144	5	H5 (1), H7(4)	0	0.04	0.00223
45	*A. neriniflorum*	Chengde, HeBei	40°58′	117°56′	357	5	H1(5)	0	0	0
46	*A. neriniflorum*	Baoshitu, JiLin	43°53′	123°31′	139	5	H5 (3), H8 (2)	0	0.6	0.00309
47	*A. neriniflorum*	Qiqihaer, HeiLongjiang	47°22′	124°12′	148	5	H5 (5)	0	0	0
48	*A. neriniflorum*	Xiwuqi, IMG	44°35′	117°35′	1,029	5	H1(5)	0	0	0
49	*A. neriniflorum*	Shipenggou, IMG	42°19′	117°50′	1,170	5	H1(2), H2(1), H3 (2)	0	0.8	0.00223
50	*A. neriniflorum*	Liulimiao, Beijing	40°37′	116°38′	743	5	H1(5)	0	0	0
51	*A. neriniflorum*	Zhenzishan, IMG	43°23′	116°39′	1,231	5	H1(5)	0	0	0
10	*A. tubiflorum*	Laojunshan, HeNan	33°43′	111°37′	2,017	10	H15 (5), H18 (5)	0	0.556	0.00024
12	*A. tubiflorum*	Longyuwan, HeNan	33°39′	111°47′	1,946	10	H18 (3), H2O (7)	1	0.467	0.0006
13	*A. tubiflorum*	Songshan, HeNan	34°30′	113°02′	1,033	10	H21 (3), H17 (7)	1	0.467	0.00081
18	*A. tubiflorum*	Guwudangshan, HeBei	36°57′	113°57′	1,399	10	H17 (10)	0	0	0
19	*A. tubiflorum*	Xiantaishan, HeNan	36°10′	113°43′	841	10	H17 (10)	0	0	0
20	*A. tubiflorum*	Longchiman, HeNan	33°42′	112°00′	1,996	10	H18 (10)	0	0	0
23	*A. tubiflorum*	Cuihuashan, ShaanXI	33°59′	109°00′	871	10	H22(10)	1	0	0
24	*A. tubiflorum*	Maijishan, GanSu	34°22′	106°01′	779	10	H15 (10)	0	0	0
25	*A. tubiflorum*	Huashan, ShaanXI	34°28′	110°05′	782	10	H15 (5), H18 (5)	0	0.556	0.00024
26	*A. tubiflorum*	Taibaishan, ShaanXI	34°03′	107°37′	975	10	H15 (1), H25(9)	1	0.2	0.00009
27	*A. tubiflorum*	Huxian, ShaanXI	33°57′	108°31′	720	10	H23(10)	1	0	0
28	*A. tubiflorum*	Lingbao, HeNan	34°30′	110°25′	870	10	H18 (5), H15 (3), H19(2)	1	0.467	0.0002
29	*A. tubiflorum*	Hujiahe, GanSu	33°43′	106°02′	934	10	H15 (9), H16(1)	1	0.2	0.00009
30	*A. tubiflorum*	Lishan, ShanXI	35°15′	111°53′	860	10	H18 (9), H24(1)	1	0.2	0.00009
33	Hybrid	Baihuashan, BeiJing	39°51′	115°38′	1,074	5	H1(2), H14(3)	0	0.6	0.00078
17	Hybrid	Shangfangshan, BeiJing	39°40′	115°49′	523	13	H14(13)	0	0	0
34	Hybrid	Daanshan, BeiJing	39°53′	115°47′	624	5	H14(5)	0	0	0
Total	*A. tubiflorum*					140	11		0.826	0.00216
Total N	*A. neriniflorum*					204	13		0.734	0.00192
Total						367	25		0.887	0.00465

### 2.2 DNA extraction, amplification and sequencing

The total genomic DNA was extracted from 100 to 150 mg dry leaves using a plant genomic DNA kit (Tiangen Biotech, Beijing, China).Three cpDNA fragments, *rps*16, *rpl*32‐*trn*L and *trn*D‐*trn*T, were sequenced using primers *rps*‐F (5′‐GTG GTA GAA AGC AACGTG CGA CTT‐3′) and ‐*rps*‐R2 (5′‐TCG GGA TCGAAC ATC AAT TGC AAC‐3′) (Endress, Queiroz, & Conti, 2006), primers *rpl*32‐F (5′‐CAG TTC CAAAAA AACGTA CTT C‐3′) and ‐*trn*L (5′‐CTGCTT CCT AAG AGC AGCGT‐3′) (Shaw, Lickey, Schilling, & Small, 2007), and primers *trn*D^GUC^F (5′‐ACC AAT TGA ACT ACA ATC CC‐3′) and *‐trn*T^GUC^ (5′‐CTA CCA CTG AGT TAA AAG GG‐3′) (Shaw et al., 2005), respectively. Polymerase chain reaction (PCR) was carried out in a 30 μl volume including Mg^2+^, dNTP, and 3 ml of buffer, with 1.5 ml of each primer, 0.3 ml of Taq polymerase and the plant DNA. The PCR parameters for the amplifications of the three primers were all: initial denaturation for 4 min at 95°C, followed by 30 cycles of denaturation (94°C, 45 s); annealing (52°C, 45 s); extension (72°C, 1 min); and a final extension for 10 min at 72°C.

The PCR products were separated in 1.5% (w/v) agarose TAE gel and purified by the Wizard PCR Preps DNA purification system (Promega, Madison, WI, USA) following the manufacturer's instructions. The cycle sequencing reactions were carried out with the purified PCR product, AmpliTaq DNA polymerase, and fluorescent Big Dye terminators. The sequencing products were generated using an ABI Prism 310 DNA sequencer (Applied Biosystems, Foster City, CA, USA) that sequenced in both directions using the PCR primers.

### 2.3 Data analysis

#### 2.3.1 DNA alignment

The cp *rps*16, *rpl32‐trn*L and *trn*D*‐trn*T were joined into one cpDNA dataset. Sequences were aligned using ClustalX ver. 1.81 (Thompson, Gibson, Plewniak, Jeanmougin, & Higgins, 1997) with subsequent manual adjustments as necessary.

#### 2.3.2 Network representation

The genealogical relationships among all the cpDNA haplotypes were examined via a median‐joining (MJ) haplotype network, which was constructed using NETWORK ver. 5.0 (Bandelt, Forster, & Rohl, 1999). In addition, to complement the results of NETWORK 5.0, we used TCS 1.21 (Clement, Posada, & Crandall, 2000) to construct haplotype relationships (Templeton, 1995). Our analysis indicated that both site mutations and indels were equally likely to evolve and that each indel originated independently of other indels.

#### 2.3.3 Phylogenetic analyses of haplotypes

To evaluate the phylogenetic relationship of the cpDNA haplotypes, maximum likelihood (ML) analyses were carried out using RAxML‐HPC BlackBox 7.7.1 (Stamatakis, 2006; Stamatakis, Hoover, & Rougemont, 2008) on the Cyber infrastructure for Phylogenetic Research (CIPRES) Science Gateway 3.3 (Miller, Pfeiffer, & Schwartz, 2010).

#### 2.3.4 Genetic structure of populations and geographical structure analyses

The number of haplotypes, haplotype diversity (*H*_d_) and nucleotide diversity (*π*), were estimated with DNASP version 6.0 (Rozas et al., 2017). The average gene diversity within populations (*H*_S_), total gene diversity (*H*_T_), and two‐population differentiation parameters (*G*_ST_ and *N*_ST_) at the species level were statistically compared using 1,000 permutations in the program Permut CpSSR 2.0 (Pons & Petit, 1996). Analyses of molecular variance (AMOVA) was applied in the program Arlequin ver 3.5 (Excoffier & Lischer, 2010) to assess the genetic differentiation within and between populations, while testing for the significance of partitions with 1,000 random permutations. The gene flow *N*_m_ among populations was obtained from the following formula: *F*_ST_ = 1/(1 + 2*N*_m_) (Wright, 1950).

#### 2.3.5 Demographic history analyses

To detect whether the population groups (all populations, *A. tubiflorum* and *A. neriniflorum*) of *A. tubiflorum* and its congener *A. neriniflorum* experienced a recent population expansion, a mismatch distribution analysis was carried out using the DNASP program (Rozas et al., 2017). A multimodal distribution of differences between the haplotypes in a given population suggests the population did not experience an expansion, whereas a unimodal distribution indicates the occurrence of an expansion. Additionally, Tajima's *D* (Tajima, 1989) and Fu & Li's D* were calculated (Fu, 1997) with DNASP.

#### 2.3.6 Bayesian divergence time estimations

Due to the lack of relevant fossil data in Allium, it is challenging to reconstruct solid divergence time estimates for *A. tubiflorum* and its congener *A. neriniflorum*. However, Bayesian analyses were used to date the tree with BEAST version 2.5 (Bouckaert et al., 2014) with a log‐normal relaxed clock based on the general substitution rates of the plastid sequence. For the cpDNA data set, because there are no available substitution rates for *Allium*, a mutation rate of 1.52 × 10^−9^ substitutions per site per year [s/s/y] proposed by Yamane, Yano, and Kawahara (2006) for chloroplast noncoding regions of grass was used to estimate the divergence times of *A. tubiflorum* and its congener *A. neriniflorum*. This mutation rate was also used by Huang, Li, Zhou, Zhou, and He (2014) to infer the evolution time of *Allium wallichii*.

The evolution model was evaluated using jModelTest 2.2.7 (Darriba, Taboada, Doallo, & Posada, 2012) and TVM+I substitution model was selected using the Akaike information criterion (AIC). 10,000,000 generations of the Markov chain Monte Carlo chains were run, sampling from every 1,000 generations. The convergence of the stationary distribution was checked by the visual inspection of plotted posterior estimates using the software Tracer version 1.7 (Rambaut, Drummond, & Xie, 2018) and that the effective sample sizes were at least 200. After discarding the first 10% trees as burn‐in, the samples were summarized in the maximum clade credibility tree using Tree Annotator version 2.5 (Bouckaert et al., 2014) with the posterior probability limit set to 0.5 and summarized common ancestor heights; and trees edited in FigTree 1.4.3 (Rambaut, 2016). In our study, three related species, *Allium prattii*,* Allium victorialis* and *Allium ovalifolium*, were selected as the outgroup based on Li et al. (2010).

#### 2.3.7 Ecological niche modeling

In addition to the sample sites from this study, 57 collection records were obtained from the Chinese Virtual Herbarium (http://www.cvh.org.cn), Specimen National Specimen Information Infrastructure (NSII, http://www.nsii.org.cn/). Based on the 57 records, the potential habitats of *A. tubiflorum* and *A. neriniflorum* were modeled with the widely used program Maxent 3.4.1 (Phillips, Miroslav, & Robert, 2018).

Nineteen climate variables based on mean values from 1970 to 2000 were downloaded from the WorldClim database (http://www.worldclim.org) (Fick & Hijmans, 2017). Variables created by Maxent are often already highly correlated (Merow, Smith, & Silander, 2013), and we excluded the variables with the Pearson correlation coefficient of *r* > 0.85. Eight variables with 30 s (~1 km^2^) spatial resolutions were retained: the mean diurnal range, isothermality, temperature seasonality, mean temperature of the driest quarter, mean temperature of the warmest quarter, annual precipitation, precipitation of the wettest month, and precipitation seasonality.

In Maxent, due to the small sample size of our data (15–79 localities), only the linear + quadratic + hinge functional forms were used (Merow et al., 2013). Here, models were built using linear + quadratic + hinge features, random training data were set to 25%, the create response curve and do jackknife options were selected, the logistic output format was logistic, and other parameters were set by default. The accuracy of the model performance was tested by the AUC of the receiver operating characteristic (ROC) (Fawcett, 2006); values above 0.7 indicate that an ecological niche modeling (ENM) is useful (Araújo, Pearson, Thuiller, & Erhard, 2005).

#### 2.3.8 Test for niche overlap

To visualize the geographical niche zone, the overlap where hybrids between *A. tubiflorum* and *A. neriniflorum* may occur, we calculated the overlapping area based on the binarized layer using ArcGIS 10.2 (ESRI, Redlands, CA, USA). We used a binary threshold from DIVA‐GIS of zero, which excluded the zero‐suitable habitats, and the minimum training presence cumulative threshold was from Maxent (Zhao, Gugger, Xia, & Li, 2016).

#### 2.3.9 Environmental variation between *Allium tubiflorum* and *Allium neriniflorum*

Three statistical approaches were used to investigate the ecological divergence between *A. tubiflorum* and *A. neriniflorum* based on the environmental variable values extracted by ArcGIS 10.2. First, principal components analysis (PCA) was performed to plot the occurrences of *A. tubiflorum* and *A. neriniflorum* in relation to the eight climate variables using Canoco 5.0 (Braak & Smilauer, 2012). Second, multivariate analysis of variance (MANOVA) was used to evaluate whether the environments differed significantly between species for the eight climate variables in SPSS 22.0 (SPSS, Chicago, IL, USA). Finally, Wilks’ *k* was used to test the null hypothesis that *A. tubiflorum* and *A. neriniflorum* had an equal mean across climate variables via discriminant function analysis (DFA) using SPSS. The significance of Wilks’ *k* was tested by chi‐square tests. If the *p*‐value of chi‐square is <0.05, a significant ecological difference between the two species can be concluded.

## 3 RESULTS

### 3.1 Haplotype distribution and phylogenetic analyses

The aligned *rps*16, *rpl*32*‐trn*L and *trn*D‐*trn*T sequences of *Allium* were 819, 888 and 777 bp in length, respectively. A 2,500‐bp data set with 36 nucleotide substitutions and 11 indels was obtained when all the three fragments were combined (Supporting Information Table S1). All the sequences were deposited in the GenBank database, with accession numbers MG709262–MG709289 (*rpl*32—*trn*L), MG709290–MG709317 (*rps*16) and MG709318–MG709345 (*trn*D—*trn*T).

Twenty‐five haplotypes (H1–H25) were detected from 30 populations of *A. neriniflorum*, 14 populations of *A. tubiflorum* and three probable hybrid populations in central and northern China (Table 1). A total of 38% and 56% of the sampled individuals carried this haplotype in *A. neriniflorum* and *A. tubiflorum*, respectively. Haplotypes H1–H13 only occurred in *A. neriniflorum*, and the other haplotypes (H15–H25) were only found in *A. tubiflorum*. Haplotypes H1–H3, H5, H7, H8, H15, H17, and H18 occurred in two or more populations, and the other haplotypes just existed in a single population (Table 1 and Figure 2). Seven haplotypes (H6, H11, H12, H16, H19, H21, and H24) were very rare, with frequencies below 2%. The most common haplotype (H1, frequency 26.2%) was present in nineteen *A. neriniflorum* populations (P1–P2, P5–P9, P14–P15, P21, P39–P42, P45, P48–P51), followed by haplotype H5 (frequency 10.1%), which was found in nine *A. neriniflorum* populations (P3–P4, P14, P37, P39, P43–P44, P46–P47); for *A. tubiflorum*, the most common haplotype was H18 (frequency 10.1%), which was identified in five *A. tubiflorum* populations (P12, P20, P25, P28, P30). The total cpDNA diversity of all populations (*H*_d_ = 0.887) was very high. The total cpDNA diversity was significantly higher in *A. tubiflorum* (*H*_d_ = 0.826) than in *A. neriniflorum* (*H*_d_ = 0.734).

A single tree was obtained by maximum likelihood analysis, and two clades were identified: clade I and clade II. Clade I consists of H1–H13, which is distributed in the northern region. Clade II include H15–H25, all occurring in the southern region (Figure 2). The two clades exactly correspond with two species.

**Figure 3:**
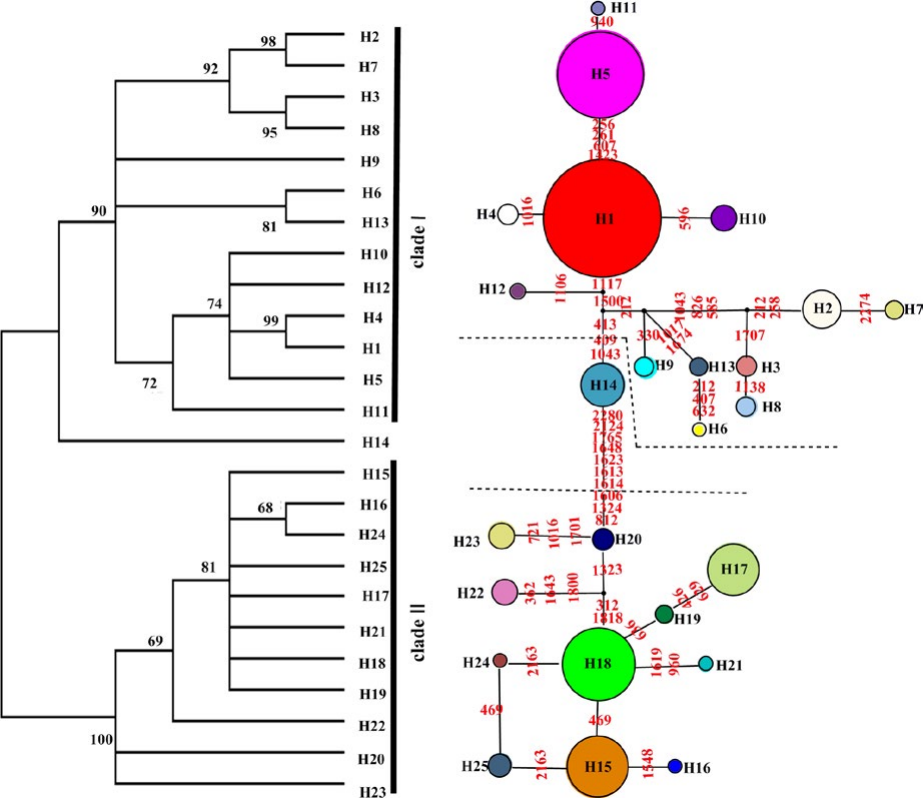
Left: maximum‐likelihood tree (no outgroup) of *Allium tubiflorum* and *Allium neriniflorum*. Bootstrap support values >50% are marked. Right: the haplotype network for *A. tubiflorum* and *A. neriniflorum*. The sizes of the circles indicate the frequency of each haplotype. Black dots indicate inferred intermediate haplotypes that were not detected in our research or were lost by extinction. The red numbers in the branch represent the mutational steps

The network analysis shows that haplotypes H1 and H18 are in the central position and form a star phylogeny with other haplotypes (Figure 3) (Sakaguchi et al., 2012). Moreover, they are widespread and predominant in most of the A. *neriniflorum* and *A. tubiflorum* populations from northern and central China, respectively, see the Figure 2. These all suggest that they represent ancestor haplotypes (Ignazio & Kohn, 2001). However, haplotype H14 is very special and become another clade, which means that populations containing H14 maybe a hybrid species. The related research is in progress.

### 3.2 Genetic diversity and genetic structure

The average gene diversity within populations (*H*_S_) is 0.265 ± 0.0427, and the total gene diversity (*H*_T_), 0.883 ± 0.0304. The coefficients of differentiation of the two species *N*_ST_ (0.844) were high and higher than *G*_ST_ (0.798) (permutation test: *p* = 0.01), indicating significant population differentiation and suggesting the existence of a phylogeographical structure (Ossa, Pérez, Armesto, & Katinas, 2013).

AMOVA analysis revealed that, in all the sampled populations, the variation attributed to differences between species was 76.22%, which is very significant, while that variation among and within population differences were 15.85% and 7.93%, respectively (Table 2). The *F*_ST_ value obtained by AMOVA also indicated high differentiation within the populations (*F*_ST_ = 0.92656, *p* < 0.001). Approximately 76% of the variation was attributed to the species differentiation, and among‐population variation accounted for just one‐quarter (16%) of the total variation (Table 3), which indicates that *A. tubiflorum* and *A. neriniflorum* are two different species. For *A. tubiflorum*, the among‐population differentiation was 87%, higher than that of its congener *A. neriniflorum* (55%). This result showed that the gene flow among populations of *A. tubiflorum* was lower than that among populations of *A. neriniflorum*. Overall, these results strongly indicate that the haplotypes are geographically structured across the species distribution range.

**Table 2:** Results of analysis of molecular variance (AMOVA) for the 46 populations of *Allium tubiflorum* and *Allium neriniflorum* based on cpDNA data (*rps*16, *rpl*32‐*trn*L and *trn*D‐*trn*T)

	Source of variation	*df*	Sum of squares	Variance components	Percentage of variation	Fixation index
Total	Between species	1	1221.657	7.27860	76.22	FSC: 0.66622*
	Among populations within species	42	475.167	1.36792	15.85	FST: 0.92656*
	Within populations	300	205.600	0.68533	7.93	FCT: 0.77997*
	Total	343	1902.424	9.33185		
*A. tubiflorum*	Among populations	13	198.557	1.50498	87.05	FST:0.87054*
	Within populations	126	28.200	0.22381	12.95	
	Total	139	226.757	1.72879		
*A. neriniflorum*	Among populations	29	276.610	1.25835	55.24	FST:0.55242*
	Within populations	174	177.400	1.01954	44.76	
	Total	203	454.01	2.27789		

*Indicated significance at *p*<0.001

**Table 3:** Mean (±*SE*) values of eight climatic variables and the significance of multivariate analysis of variance (MANOVA) and Wilks’ *λ*, as determined by discriminant function analysis (DFA)

Variables	*Allium neriniflorum*	*Allium tubiflorum*
Mean diurnal range**	11.304 ± 0.319	9.531 ± 0.433
Isothermality***	24.689 ± 0.455	28.901 ± 0.619
Temperature seasonality***	1249.198 ± 24.693	853.149 ± 33.586
Mean temperature of the driest quarter***	−10.029 ± 0.710	−1.206 ± 0.966
Mean temperature of the warmest quarter#	20.664 ± 0.359	20.031 ± 0.489
Annual precipitation***	459.649 ± 20.774	737.700 ± 28.215
Precipitation of the wettest month#	137.243 ± 4.757	150.600 ± 6.470
Precipitation seasonality***	114.365 ± 1.613	79.294 ± 2.195
Wilks’ *λ* ***		0.103 (*χ* ^2^ = 115.885)

*, ** and ***Indicate the value is significant at *p* < 0.05, *p* < 0.01, and *p* < 0.001, respectively, and ^#^indicates the value is not significant (*p* > 0.05) based on MANOVA.

Cp DNA data shows that *A. tubiflorum* had higher levels of genetic diversity based on cpDNA (*H*_d_ = 0.826) than *A. neriniflorum* (*H*_d_ = 0.734) (Table 1). Moreover, the genetic differentiation among *A. tubiflorum* populations (*F*_ST_ = 0.87) was also far higher than that among *A. neriniflorum* populations (*F*_ST_ = 0.55). Accordingly, the calculated gene flow among *A. tubiflorum* populations (*N*_m_ = 0.07) was lower than that among *A. neriniflorum* populations (*N*_m_ = 0.40).

### 3.3 Demography

**Figure 4:**
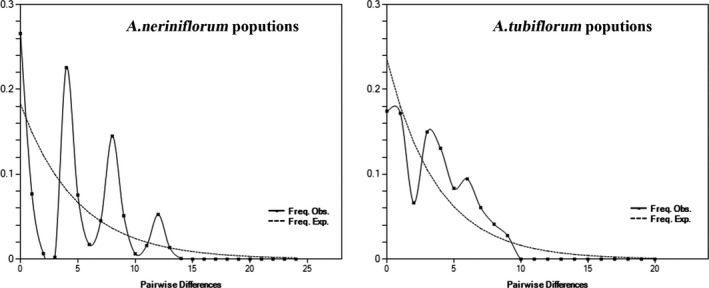
Mismatch distribution plots for the haplotypes of *Allium tubiflorum* and *Allium neriniflorum*. The solid line shows observed values, whereas the dashed line represents expected values under a mode of sudden (stepwise) population expansion

To test whether population groups of *A. neriniflorum* and *A. tubiflorum* underwent a recent demographic population expansion, we plotted the mismatch distribution of each group based on the observed number of differences between pairs of haplotypes. The mismatch distributions of the two species were clearly multimodal curves (Figure 4). The test values for neutrality of the total dataset (*A. neriniflorum*: Tajima's *D* = 0.26974, *p* > 0.10; Fu and Li's D* = 0.95285, *p* > 0.10; *A. tubiflorum*: Tajima's *D* = 0.15862, *p* > 0.10; Fu and Li's D* = 1.12033, *p* > 0.10) were significantly positive values, which confirmed that the *A. neriniflorum* and *A. tubiflorum* populations had not undergone a recent expansion.

### 3.4 Divergence time estimates

**Figure 5:**
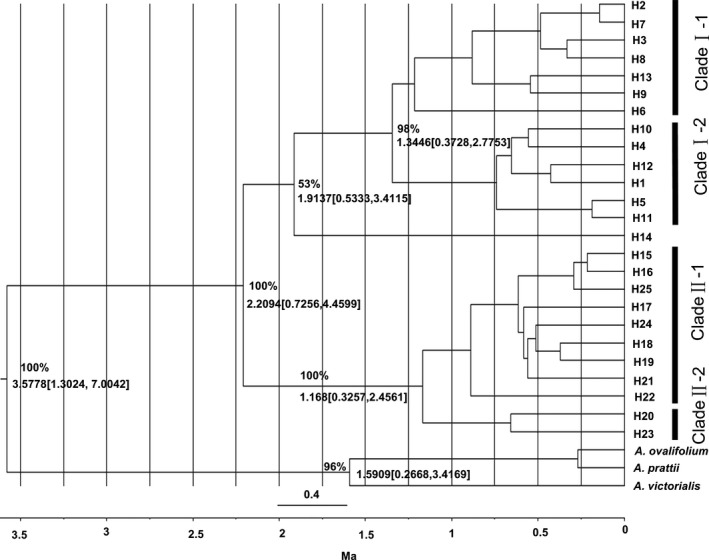
The phylogenetic relationships determined by Bayesian inference for cpDNA haplotypes 1–25 based on BEAST 2.5. The numbers above the branches show the posterior probability and divergence time values (only the major clades)

Haplotypes systemic tree was constituted by the Bayesian analysis and the divergent time of the conjunct ancestors about *A. neriniflorum* and *A. tubiflorum* was inferred using BEAST 2.5 (Figure 5). Divergence time estimated between the two species occurred in approximately 2.2094 Ma, in the early Pleistocene epoch. Moreover, we deduced *A. neriniflorum* were divided into two lineages in approximately 1.3446 Ma and *A. tubiflorum*,* in* approximately 1.168 Ma. The intraspecific divergence time of two species is also in the early Pleistocene epoch.

### 3.5 Ecological niche modeling

**Figure 6:**
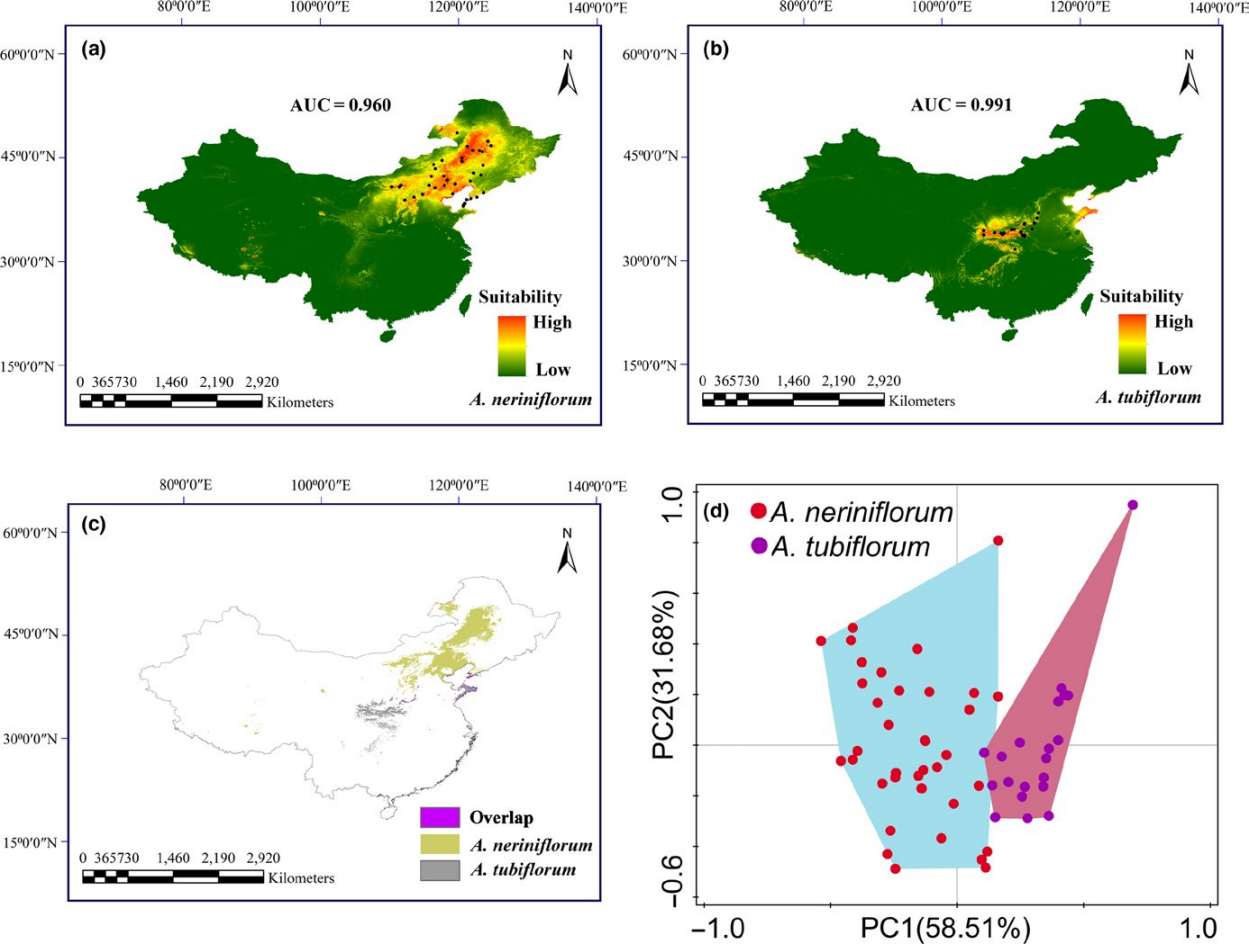
Potential habitats and ecological divergence of *Allium neriniflorum* and *Allium tubiflorum* estimated by Maxent (a–c) and principal components analysis (PCA) (d). Black points show occurrences of *A. neriniflorum* and *A. tubiflorum*. The area under the curve (AUC) >0.90 indicates the model performed well. The ecological separation between *A. neriniflorum* (yellow) and *A. tubiflorum* (gray) are shown; violet indicates the possible hybrid zone (c). The variations represented by PC1 and PC2 are 58.51% and 31.68%, respectively; *A. neriniflorum* (red) and *A. tubiflorum* (violet) are separated by the PCs

The AUC value for the current potential distribution of *A. neriniflorum* and *A. tubiflorum* both were relatively high (AUC > 0.9), demonstrating a reliable predictive model performance. Model exhibited distinctive ecological niches between *A. neriniflorum* and *A. tubiflorum* (Figure 6a–c). PCA biplots displayed distinct habitats between *A. neriniflorum* and *A. tubiflorum* (Figure 6d). The bootstrapping MANOVA estimates indicated that most climate variables contributed significantly to niche divergence, except for the mean temperature of the warmest quarter and the precipitation of the wettest month (Table 3). Wilks’ *k* indicated a significant climatic difference between the two species habitats (*χ* ^2^ = 115.885, *p* < 0.001) (Table 3).

## 4 DISCUSSION

### 4.1 Genetic characteristics of populations of two species

Our cpDNA data support the validity of both species, *A. neriniflorum* and *A. tubiflorum*, although both species show close morphological similarities (Lu et al., 2016). Therefore, cp DNA can be used as a marker to distinguish them in plant taxonomy.

We also found some differences that *A. tubiflorum* had higher levels of genetic diversity and the lower gene flow compared to *A. neriniflorum*. There are three possible reasons for these differences. One may be related to the plant's life history traits. The longer life history of *A. tubiflorum* may contribute to the creation and maintenance of its high level of genetic variability (Jian, Tang, Zhong, & Shi, 2010). The second reason may be the topographical differences in the ecological environments of *A. tubiflorum* and *A. neriniflorum*. In our sites, *A. tubilflorum* was limited to the rock crevices of mountains and slop places with low levels of human activities from 550 to 2,050 m above sea level, rarely plains and valleys. These fragmented living conditions prevent the flow of genes among the distance‐isolated populations of *A. tubiflorum*. However, *A. neriniflorum* individuals were commonly found in wide and successive ranges such as meadows, sandy places and the mountains near sea level at an altitude of 100–1,500 m. These less blocked ecological environments resulted in frequent gene flow among populations. Therefore, we can inferred that the mountain taxa showed a high biodiversity, which agrees with the “mountain‐geo‐biodiversity hypothesis”. The third reason may be the differently configured features of species itself. We found that *A. tubiflorum* has 0.8–7 cm pedicels and 3–6 ovules per locule, while *A. neriniflorum* has 4.5–11 cm pedicels and 5–8 ovules per locule. From the above data, the shorter pedicels and lower number of ovules per locule of *A. tubiflorum* cause their seeds to spread a shorter distance than those of *A. neriniflorum*. The population genetic differences of monolepsis cpDNA reflect the pathways and distance of species seeds, as was also shown by the study of Gao and Ge (1999)

### 4.2 Multiple refugia

Phylogeographic studies on cool temperate deciduous species, such as *Ostryopsis davidiana* (Tian et al., 2009), *Mongolian oak* (Zeng, Wang, Liao, Wang, & Zhang, 2015), and *Juglans mandshurica* (Bai, Liao, & Zhang, 2010), suggested the in situ survival of species and multiple refugia in the northern regions of modern distribution of temperate forests. As herbaceous plants sensitive to climate, did *A. neriniflorum* and *A. tubiflorum* survive in a similar way as the abovementioned species? The cpDNA phylogenetic relationship and haplotype network analyses of the two species indicated that they belong to two different lineages restricted to the northeastern and central areas of China (Figure 3). The genetic differentiation between these two areas was high. Moreover, mismatch distribution confirmed that populations from the two regions had not undergone recent expansions. These data are indicative of fragmentation events and the existence of at least two glacial refugia in the current range of the *Allium*s species (Aoki, Matsumura, Hattori, & Murakami, 2006; Chen et al., 2012).

Moreover, the network analysis shows that haplotypes H1 and H18 are ancestor haplotypes. If ancestral and derived haplotypes do not overlap and are located in different regions, then ancestral haplotypes should be found close to refugia, while derived haplotypes are more likely to occur at the leading edge of the range expansion (Hewitt, 2000). In *A. tubiflorum*, haplotypes H17 and H15 are distributed in the northeastern and southwestern leading edges, respectively, whereas the ancestral haplotype H18 is restricted to the six populations (P10, P12, P20, p25, p28, p30) in the edge of the Taihang Mountains and Qinling Mountains (Figure 2), the possible range of the refugium for *A. tubiflorum* during the glaciation. This coincides with the sanctuary of temperate walnut tree and temperate deciduous shrub species (*Ostryopsis* davidiana Decne., Betulaceae) (Tian, 2007). For *A. neriniflorum*, our study found the ancestral haplotype H1 had a disjunctive distribution in the northernmost edge (population P14) and central region (population P49, P2) of the range (Figure 2). Moreover, the disjunctive distance was nearly 500 km. The proposal is that, in the past, haplotype H1 was widely dispersed in the range of the examined populations and later was replaced by other haplotypes in the middle areas of the studied range owing to the continuous contraction and colonization during the Quaternary glacial and interglacial periods. Under this proposal, population P14 in Lamadianzi (HeiLongjiang), a very damp grass lake with relatively steady climatic conditions, may represent a cryptic northern refugia during the glacial period (Li, Shao, Lu, Zhang, & Qiu, 2012). Additionally, the ENM analyses indicated that these regions were suitable distribution ranges for the two species, which also supported the possibility of these refugia.

### 4.3 Evolution of two species

To investigate what paleoclimatic events triggered the divergence of *A. neriniflorum* and *A. tubiflorum*, the divergence time was estimated based on genetic distances (Figure 5). Our results show a population divergence of *A. neriniflorum* and *A. tubiflorum* at approximately 2.2094 Ma, which is coincident with the onset of the Quaternary glaciation (approximately 3–2 Ma). In this period, Taihang Mountains nearly formed a S‐N‐striking uplifted mountain range within the central North China block. However, the divergence time is different from that in Hauenschild et al. (2017), which may result from the different analysis methods. Our analyses is as solid as theirs, and that even if our results would be slightly older, our interpretation remains similar and plausible.

Our ENM analysis suggests that *A. neriniflorum* and *A. tubiflorum* each occupy a distinct climate niche (Figure 6). Niche differentiation can provide the preconditions for the adaptive divergence of fragmented populations and subsequent speciation (Abbott & Brennan, 2014; Hewitt, 1996; Zhao et al., 2016). Speciation can occur largely as an outcome of ecological differentiation maintaining morphological differences, even after substantial gene influx between related species (Mckinnon et al., 2004; Nagy, 1997; Zhao et al., 2016).

We inferred that the populations of ancestral taxon about *A. tubiflorum* and *A. neriniflorum* were once widely distributed in north and central China in the warm period of the Pliocene. Then, a cooling period occurred from the Pliocene to the Pleistocene. Especially in the Quaternary glaciation, the climate became colder and drier, and there was an obvious alternation between glacial–interglacial cycles (Crippa et al., 2016; Tian, Wei, Cai, Wang, & Li, 2018). In glacial epoch, the ancestral populations began to contract and were limited in some fragmented refugia. While in interglacial epoch, Taihang Mountain became an expansion barrier between the populations in north China and in central China. The pattern differentiation occurred owing to adaptation to different ecological environments and climates. At last, a new species, *A. neriniflorum*, was formed and fixed as a result of adaptation. NJ network analysis also revealed that populations of *A. neriniflorum* could be further divided into two distinctive groups with high bootstrap values of 99% (Clade I‐1, Clade I‐2) at approximately 1.3446 Ma. Almost simultaneously, the intraspecific divergence of *A. tubiflorum* occurred at approximately 1.168 Ma. These data show that the simultaneous intraspecific differentiation of *A. tubiflorum* and *A. neriniflorum* resulted from common climate events in the Quaternary (Yang, Dong, & Lei, 2009). Six great glaciations occurred during the Quaternary glaciation in China (Cui et al., 2011). It was previously unclear which glaciations had the greatest impact on the current vegetation of *A. tubiflorum* and *A. neriniflorum*. The intraspecific divergence time of the two species coincides with the time of the first glaciation (approximately 1.2 Ma), in the late stage of the Early Pleistocene (Cui et al., 2011). Therefore, it is most likely that the divergence of the main lineages of *A. tubiflorum* and *A. neriniflorum* can be attributed to the climate oscillation of the largest glaciation in the Quaternary (Zhang, Fengquan, & Jianmin, 2000; Zheng, Xu, & Shen, 2002). In a word, the evolution of *A. neriniflorum* and *A. tubiflorum* mainly resulted from the violent climate changes that occurred in the Quaternary glaciation.

## CONFLICT OF INTEREST

None declared.

## AUTHOR CONTRIBUTIONS

JTY and XJH designed the experiment. JTY collected the field data. JTY, SDZ, and DQH analyzed the data. JTY drafted the text. All authors revised it critically and significantly improved the text.

## DATA ACCESSIBILITY

DNA sequences: Genbank accessions MG709262–MG709345. Climate data and MaxEnt input files, morphological data, Sampling locations: Figshare. https://doi.org/10.6084/m9.figshare.6224939.
