# Supplementary material for: Phylogeography of two closely related species of Allium endemic to East Asia: Population evolution in response to climate oscillations
Source: Ecol Evol. 2018 Jul 16;8(16):7986–99. doi: 10.1002/ece3.4338 (PMC6145274; doi:10.1002/ece3.4338)
Supplement: Supplementary file 1 [file ECE3-8-7986-s001.docx]

- *Ecology and Evolution*
- **SUPPORTING INFORMATION**

**Phylogeography of two closely related species of *Allium* endemic to East Asia:**

**Population evolution in response to climate oscillations**

Jingtian Yang  | Songdong Zhou | Deqing Huang | Xingjin He

**Supplementary table (Table S1)**

Table S1 Distributions of variable sites of each haplotype for *A. tubiflorum*, *A. neriniflorum and A. peikingense* in the rps16 (1-819), rpl32-trnL (820-1707) and trnD-trnT (1708-2484) spacers.

|  | Nucleotide position | | | | | | | | | | | | | | | | | | | | | | | | | | | | | | | |
| --- | --- | --- | --- | --- | --- | --- | --- | --- | --- | --- | --- | --- | --- | --- | --- | --- | --- | --- | --- | --- | --- | --- | --- | --- | --- | --- | --- | --- | --- | --- | --- | --- |
|  | rps16(1-819) | | | | | | | | | | | | | | | | | rpl32-trnL (820-1707) | | | | | | | | | | | | | | |
| Haplotype | 212 | 256 | 258 | 261 | 330 | 381 | 426 | 432 | 442 | 488 | 604 | 615 | 648 | 651 | 655 | 723 | 762 | 853 | 867 | 981 | 1001 | 1057 | 1075 | 1118 | 1195 | 1214 | 1247 | 1432 | 1433 | 1532 | 1615 | 1663 |
| H1 | T | T | A | T | - | - | A | T | G | C | A | T | T | T | - | - | T | G | G | G | T | T | - | e | f | g | G | A | - | - | C | C |
| H2 | G | T | C | T | - | - | T | T | G | C | G | T | T | T | - | - | T | G | A | G | T | T | - | - | f | - | G | A | - | - | A | C |
| H3 | A | T | A | T | - | - | A | T | G | C | G | T | T | T | - | - | T | G | A | G | T | T | - | - | f | - | G | A | - | - | A | C |
| H4 | T | T | A | T | - | - | A | T | G | C | A | T | T | T | - | - | T | G | G | G | T | - | - | e | f | g | G | A | - | - | C | C |
| H5 | T | A | A | A | - | - | A | T | G | C | A | T | T | T | - | c | T | G | G | G | T | T | - | e | f | g | G | A | - | h | C | C |
| H6 | T | T | A | T | - | - | T | T | G | C | A | T | T | A | - | - | T | G | G | G | T | T | d | e | f | - | G | A | - | - | A | C |
| H7 | G | T | C | T | - | - | T | T | G | C | G | T | T | T | - | - | T | G | A | G | T | T | - | - | f | - | G | A | - | - | A | C |
| H8 | A | T | A | T | - | - | A | T | G | C | G | T | T | T | - | - | T | G | A | G | T | T | - | - | f | - | T | A | - | - | A | C |
| H9 | A | T | A | T | a | - | A | T | G | C | A | T | T | T | - | - | T | G | G | G | T | T | - | e | f | - | G | A | - | - | A | C |
| H10 | T | T | A | T | - | - | A | T | G | C | A | G | T | T | - | - | T | G | G | G | T | T | - | e | f | g | G | A | - | - | C | C |
| H11 | T | A | A | A | - | - | A | T | G | C | A | T | T | T | - | c | T | G | G | T | T | T | - | e | f | g | G | A | - | h | C | C |
| H12 | T | T | A | T | - | - | A | T | G | C | A | T | T | T | - | - | T | G | G | G | T | T | - | e | - | - | G | A | - | - | C | C |
| H13 | A | T | A | T | - | - | A | T | G | C | A | T | T | T | - | - | T | G | G | G | T | T | d | e | f | - | G | A | - | - | A | C |
| H14 | T | T | A | T | - | - | A | C | G | A | A | T | T | T | - | - | T | G | G | G | T | T | - | - | f | - | G | A | - | - | A | C |
| H15 | G | T | A | T | - | - | A | C | G | C | A | T | T | T | - | - | T | A | G | G | T | T | - | - | f | - | G | T | T | - | A | C |
| H16 | G | T | A | T | - | - | A | C | G | C | A | T | T | T | - | - | T | A | G | G | T | T | - | - | f | - | G | T | T | - | A | A |
| H17 | G | T | A | T | - | - | A | C | T | A | A | T | C | T | b | - | T | A | G | G | T | T | - | - | f | - | G | T | T | - | A | C |
| H18 | G | T | A | T | - | - | A | C | G | A | A | T | T | T | - | - | T | A | G | G | T | T | - | - | f | - | G | T | T | - | A | C |
| H19 | G | T | A | T | - | - | A | C | G | A | A | T | T | T | b | - | T | A | G | G | T | T | - | - | f | - | G | T | T | - | A | C |
| H20 | T | T | A | T | - | - | A | C | G | A | A | T | T | T | - | - | T | A | G | G | T | T | - | - | f | - | G | A | T | - | A | C |
| H21 | G | T | A | T | - | - | A | C | G | A | A | T | T | T | - | - | T | A | G | G | G | T | - | - | f | - | G | T | T | - | A | C |
| H22 | T | T | A | T | - | T | A | C | G | A | A | T | T | T | - | - | T | A | G | G | T | T | - | - | f | - | G | T | T | - | A | C |
| H23 | T | T | A | T | - | - | A | C | G | A | A | T | T | T | - | - | G | A | G | G | T | G | - | - | f | - | G | A | T | - | A | C |
| H24 | G | T | A | T | - | - | A | C | G | A | A | T | T | T | - | - | T | A | G | G | T | T | - | - | f | - | G | T | T | - | A | C |
| H25 | G | T | A | T | - | - | A | C | G | C | A | T | T | T | - | - | T | A | G | G | T | T | - | - | f | - | G | T | T | - | A | C |

The small letters denote different insertions： a (330-349), TAGAAAGAAATAATAAAAAA; b (655-662), ATGTTTTT; c (723-738), TTACATTTA ATATA TT;d(1075-1092), TAATGTAATTACTAAATT; e (1118-1132), AATTAGAAATTAAAA; f (1195-1203), AATTATTCT; g (1214-1226), TCTAA TTATTCCA; h (1532-1538), CTAATCT; i (1758-1766), TAATGGAAA; j (1830-1850), CAGAATATAATCCTATAGAAT; k (1943-1957), CATT ATAGTATGATG.

| Nucleotide position | | | | | | | | | | | | | | | |
| --- | --- | --- | --- | --- | --- | --- | --- | --- | --- | --- | --- | --- | --- | --- | --- |
| trnD-trnT (1708-2484) | | | | | | | | | | | | | | | |
| Haplotype | 1  7  2  1 | 1  7  2  9 | 1  7  3  0 | 1  7  3  4 | 1  7  3  8 | 1  7  5  8 | 1  7  7  1 | 1  7  9  7 | 1  8  3  0 | 1  9  0  8 | 1  9  4  3 | 2  2  8  1 | 2  3  2  0 | 2  4  3  1 | 2  4  3  7 |
| H1 | A | G | T | T | C | i | A | G | j | C | - | T | G | A | G |
| H2 | A | G | T | T | C | i | A | G | j | C | - | T | G | A | G |
| H3 | A | G | T | T | C | i | A | G | G | C | - | T | G | A | G |
| H4 | A | G | T | T | C | i | A | G | j | C | - | T | G | A | G |
| H5 | A | G | T | T | C | i | A | G | j | C | - | T | G | A | G |
| H6 | A | G | T | T | C | i | A | T | j | C | - | T | G | A | G |
| H7 | A | G | T | T | C | i | A | G | j | C | - | T | G | C | G |
| H8 | A | G | T | T | C | i | A | G | G | C | - | T | G | A | G |
| H9 | A | G | T | T | C | i | A | G | j | C | - | T | G | A | G |
| H10 | A | G | T | T | C | i | A | G | j | C | - | T | G | A | G |
| H11 | A | G | T | T | C | i | A | G | j | C | - | T | G | A | G |
| H12 | A | G | T | T | C | i | A | G | j | C | - | T | G | A | G |
| H13 | A | G | T | T | C | i | A | T | j | C | - | T | G | A | G |
| H14 | A | G | T | T | C | i | A | G | j | C | - | T | G | A | G |
| H15 | G | A | C | T | T | i | T | G | j | A | - | G | G | A | A |
| H16 | G | A | C | T | T | i | T | G | j | A | - | G | G | A | A |
| H17 | G | A | C | T | T | i | T | G | j | A | - | G | G | A | A |
| H18 | G | A | C | T | T | i | T | G | j | A | - | G | G | A | A |
| H19 | G | A | C | T | T | i | T | G | j | A | - | G | G | A | A |
| H20 | G | A | C | T | T | i | G | G | j | A | - | G | G | A | A |
| H21 | G | A | C | C | T | i | T | G | j | A | - | G | G | A | A |
| H22 | G | A | C | T | T | - | G | G | j | A | k | G | G | A | A |
| H23 | G | A | C | T | T | i | G | G | G | A | - | G | G | A | A |
| H24 | G | A | C | T | T | i | T | G | j | A | - | G | T | A | A |
| H25 | G | A | C | T | T | i | T | G | j | A | - | G | T | A | A |
